# Artificial Intelligence Predicts Severity of COVID-19 Based on Correlation of Exaggerated Monocyte Activation, Excessive Organ Damage and Hyperinflammatory Syndrome: A Prospective Clinical Study

**DOI:** 10.3389/fimmu.2021.715072

**Published:** 2021-08-27

**Authors:** Olga Krysko, Elena Kondakova, Olga Vershinina, Elena Galova, Anna Blagonravova, Ekaterina Gorshkova, Claus Bachert, Mikhail Ivanchenko, Dmitri V. Krysko, Maria Vedunova

**Affiliations:** ^1^ Upper Airways Research Laboratory, Department of Head and Skin, Ghent University, Ghent, Belgium; ^2^ Institute of Biology and Biomedicine, National Research Lobachevsky State University of Nizhniy Novgorod, Nizhniy Novgorod, Russia; ^3^ Institute of Information Technology, Mathematics and Mechanics, National Research Lobachevsky State University of Nizhniy Novgorod, Nizhniy Novgorod, Russia; ^4^ Privolzhsky Research Medical University, Nizhny Novgorod, Russia; ^5^ Cell Death Investigation and Therapy Laboratory, Department of Human Structure and Repair, Ghent University, Ghent, Belgium; ^6^ Department of Pathophysiology, Sechenov First Moscow State Medical University (Sechenov University), Moscow, Russia; ^7^ Cancer Research Institute, Ghent, Belgium

**Keywords:** COVID-19, prediction models, artificial intelligence, IL-6, macrophage derived cytokine

## Abstract

**Background:**

Prediction of the severity of COVID-19 at its onset is important for providing adequate and timely management to reduce mortality.

**Objective:**

To study the prognostic value of damage parameters and cytokines as predictors of severity of COVID-19 using an extensive immunologic profiling and unbiased artificial intelligence methods.

**Methods:**

Sixty hospitalized COVID-19 patients (30 moderate and 30 severe) and 17 healthy controls were included in the study. The damage indicators high mobility group box 1 (HMGB1), lactate dehydrogenase (LDH), aspartate aminotransferase (AST), alanine aminotransferase (ALT), extensive biochemical analyses, a panel of 47 cytokines and chemokines were analyzed at weeks 1, 2 and 7 along with clinical complaints and CT scans of the lungs. Unbiased artificial intelligence (AI) methods (logistic regression and Support Vector Machine and Random Forest algorithms) were applied to investigate the contribution of each parameter to prediction of the severity of the disease.

**Results:**

On admission, the severely ill patients had significantly higher levels of LDH, IL-6, monokine induced by gamma interferon (MIG), D-dimer, fibrinogen, glucose than the patients with moderate disease. The levels of macrophage derived cytokine (MDC) were lower in severely ill patients. Based on artificial intelligence analysis, eight parameters (creatinine, glucose, monocyte number, fibrinogen, MDC, MIG, C-reactive protein (CRP) and IL-6 have been identified that could predict with an accuracy of 83−87% whether the patient will develop severe disease.

**Conclusion:**

This study identifies the prognostic factors and provides a methodology for making prediction for COVID-19 patients based on widely accepted biomarkers that can be measured in most conventional clinical laboratories worldwide.

## Introduction

The recent emergence of a novel, pathogenic SARS-coronavirus 2 (SARS-CoV-2) in China and its rapid spread caused a global COVID-19 pandemic affecting more than 165 million people worldwide. SARS-CoV-2 infects host cells by binding to angiotensin-converting enzyme 2 (ACE-2) receptor primed by host transmembrane serine protease 2 (TMPRSS2), a multidomain type II transmembrane serine protease of the hepsin/TMPRSS subfamily (1). After passing the initial replication stage, SARS-CoV-2 causes a disease of varied severity. The disease varies from an asymptomatic condition in children, teenagers and young adults to a severe, lethal disease in the elderly (2). COVID-19 infection in a susceptible person can cause hyperinflammatory syndrome induced by the inappropriate triggering of danger sensing accompanied by cytokines and chemokines release, complement activation, and potentially life-threatening failure of respiratory, renal and hepatic systems, which can lead to death (3–7).

Importantly, 14−17% of COVID-19 patients develop a severe form of the disease requiring oxygen support and admission to the intensive care unit (ICU) (8–10). Underlying medical conditions such as diabetes, chronic cardiac diseases, chronic kidney diseases and obesity contribute to the severity of COVID-19 (11, 12). Moreover, it was recently shown that genetic factors could predispose to severe disease, including DNA polymorphisms in ACE2, TMPRSS2 (13) or HLA-I genotype (14). The COVID Human Genetic Effort has identified mutations in the type I interferon (IFN) pathway that may account for 14% of severe COVID-19 cases (2). In this regard, production of type I IFN is defective in severe COVID-19 patients (15). However, the severity of the disease in a large group of patients cannot be explained only by genetic predisposition. Various combinations of inflammatory cytokines and biochemical factors have been shown to be typical in more severe COVID-19. For example, IL-6, CRP and Krebs von den Lungen-6 (KL-6) together have been shown to be indicators of the severity of COVID-19 (16). Patients admitted to ICU had higher levels of IL-6, CRP and procalcitonin (17). Moreover, in severe cases, lymphopenia and higher levels of ALT, LDH, CRP, ferritin and D-dimer have been detected, as well as higher levels of IL-6, IL-10, IL-2RA and TNF-α (2).

Prediction of the severity of COVID-19 at its onset is important for providing adequate and timely management to reduce mortality. A combination of cytokines has been shown by using unsupervised principal component analysis to predict different degrees of severity of COVID-19. That analysis has shown the key roles of TNF-α, IL-6, IL-8, IL-1β and type I IFNs in patients undergoing extracorporeal membrane oxygenation (ECMO) (18). Notably, less seriously affected patients have been characterized by a type I IFN response, with increased IFN-α and IFN-β (18). That finding reconfirmed earlier studies showing that inborn errors in type I IFN response could underlie the lethality COVID-19 (19). Moreover, a combination of factors has been shown to be predictive of increased mortality. Laguna-Goya et al. have demonstrated that high IL-6, CRP, LDH, ferritin, D-dimer, neutrophil count, and neutrophil-to-lymphocyte ratio are all predictive of mortality (20). When a machine learning-based model was applied, CRP, age, LDH, ferritin and IL-10 turned out to be predictors of COVID-19 related mortality (21).

Severely ill COVID-19 patients often develop multiorgan damage, including in liver (22), kidney (23) and heart (24); this damage was associated with coagulation abnormalities and thrombosis (25). It is well known that virally infected or dying cells emit endogenous damage-associated molecular pattern molecules (DAMPs), which serve as danger signals. These molecules have non-immunological functions inside viable cells but their emission by dead or damaged cells triggers an immune response (26). HMGB1, one of the most extensively studied DAMPs, is correlated with the severity of tissue damage in patients with numerous lung disorders, including severe pneumonia (27). In a recent study, COVID-19 patients admitted to ICU had higher levels of HMGB1 compared to healthy controls (28). Several other damage molecules, such as LDH, AST and ALT, are often associated with multiorgan damage and might be used to estimate the severity of COVID-19 infection (29). However, it is difficult to predict disease severity in a large group of patients, and a more complex multifactorial analysis and prediction methods are needed to predict the development of severe disease upon hospitalization in order to initiate early treatment and possibly achieve better outcomes. Therefore, in the current study, we examined tissue damage markers such as HMGB1, LDH, AST, ALT and blood coagulation parameters, in combination with the profile of 47 cytokines, and analyzed them by unbiased machine learning methods (Logistic Regression, Support Vector Machine and Random Forest) to identify a combination of factors that could help to predict severe COVID-19.

Machine learning algorithms are widely used in medicine, including the study of COVID-19 (30). Recent studies using machine learning are devoted to assessing disease severity in COVID-19 patients based on blood and urine tests (31), determination of cytokine profiles associated with the severity and mortality of patients with COVID-19 (18), development of prognostic models for predicting mortality of patients with COVID-19 (32, 33), analysis of chest computed tomography (CT) scans (34), identification of novel drug candidates against COVID-19 (35), and many others. In this study we investigated the possibility of predicting the severity of COVID-19 by using cytokines/blood test data. To solve the binary classification problem, we built a logistic regression model, named Support Vector Machine and Random Forest, which is widely used to construct clinical prediction models (36–38).

## Methods

### Patients

This prospective study was performed at the University Clinic of Privolzhsky Research Medical University, Nizhny Novgorod, Russia. It was conducted in accordance with the Declaration of Helsinki and approved by the local ethics committee of Nizhny Novgorod State University. Male and female patients aged 18−85 years old were included in the study on day 1−3 of hospitalization. All participating patients provided written, informed consent. Pregnant women and patients with severe immunodeficiency were excluded. Only hospitalized patients in whom the presence of SARS-CoV-2 in pharyngeal swabs was determined by real-time reverse-transcription polymerase chain reaction (RT-PCR). Between May 2020 and August 2020, 60 COVID-19 patients were enrolled. The healthy controls (n=17) were contact patients, who had no complaints and were tested negative by an antigen RT-PCR test. The demographic characteristics of the patients are provided in **Table 1**. The diagnosis of COVID-19 and treatment were made according to the Ministry of Health of Russian Federation “Temporary guidelines on the prevention, diagnosis and treatment of COVID-19” version 7.0 (39). The patients were separated into two groups according to the severity of pulmonary involvement and the need for oxygen support. Oxygen was supplied through masks but four patients were mechanically ventilated. Peripheral blood samples were taken on the day of hospitalization, during the second week, and in some cases during week seven. The control group was represented by healthy volunteers of the corresponding age without acute viral infection.

**Table 1:** Demographics and baseline characteristics of patients included in the study.

Demographic characteristics	Controls	Moderate	Severe	p -value
	(n = 17 )	(n = 30)	(n = 30)	
Age, years	54,7 ± 19,7	47,7 ± 16,2	59,1 ± 15,9	0,0445*
Gender	7M/10F	7M/23F	11M/19F	
Body mass index	25,09 ± 3,6	27,8 ± 4,7	31,6 ± 4,9	0,0228*, 0,0001^#^
SpO2 at admission (%)	97 ± 1,5	95 ± 1,9	89,9 ± 5,3	<0,0001*, <0,0001^#^
Respiratory rate (breaths per min)	18 ± 2	20 ± 2	25 ± 6	<0,0001*, <0,0001^#^
Before admission to the hospital, days	0	9,1 ± 6	9 ± 3,6	ns
Admission to the ICU, days	0	0	15	
Oxygen support (n,%)	0 (0)	0 (0)	15 (50%)	
Deaths (n,%)	0 (0)	0 (0)	4 (13%)	
**Symptoms at admission**				
Fever (n,%)				
>38°C	0 (0)	17 (56%)	22 (73%)	
<38°C	0 (0)	4 (13%)	1 (3%)	
Cough (n,%)	0 (0)	19 (63%)	16 (53%)	
Fatigue (n,%)	0 (0)	24 (80%)	25 (83%)	
Shortness of breath (n,%)	0 (0)	16 (53%)	23 (76%)	
Anosmia (n,%)	0 (0)	17 (56%)	9 (30%)	
Chest pain (n,%)	0 (0)	14 (46%)	12 (40%)	
Headache (n,%)	0 (0)	16 (53%)	12 (40%)	
Myalgia (n,%)	0 (0)	10 (33%)	8 (26%)	
Rhinorrhea (n,%)	0 (0)	7 (23%)	10 (33%)	
Throat pain (n,%)	0 (0)	6 (20%)	6 (20%)	
Diarrhea (n,%)	0 (0)	5 (16%)	6 (20%)	
Hemoptysis (n,%)	0 (0)	1 (3%)	0 (0%)	
**Comorbidity**				
Any comorbidity (n,%)	7 (41%)	18 (60%)	25 (83%)	
Hypertension (n,%)	4 (23%)	16 (53%)	24 (80%)	
Diabetes (n,%)	1 (6%)	1 (3%)	13 (43%)	
Cardiovascular disease (n,%)	3 (17%)	4 (13%)	14 (46%)	
Malignancy (n,%)	0 (0)	4 (13%)	5 (16%)	
Stroke (n,%)	0 (0)	1 (3%)	3 (10%)	
Chronic lung diseases (n,%)	0 (0)	2 (6%)	2 (6%)	
Arrhythmia (n,%)	0 (0)	4 (13%)	1 (3%)	
Rheumatoid arthritis (n,%)	1 (6%)	0 (0%)	1 (3%)	
Smoking (n,%)	1 (6%)	1 (3%)	0 (0%)	

*between moderate and severe, ^#^between controls and severe. The information on the patients included in the study is provided. The number (n) of the patients in each group is provided with description of their symptoms at admission and comorbidities. For demographic characteristics data are presented as median ± standard deviation. P values comparing the groups of healthy controls, moderate and severe cases are produced by comparison of the data for normal (Gaussian) distribution (alpha = 0.05) using D’Agostino & Pearson test. The normally distributed data were analyzed by the one-way ANOVA with Dunnett’s multiple comparisons test. The data, which were not normally distributed, were analyzed by an ANOVA Kruskal-Wallis test with Dunn’s test for multiple comparisons, ns stands for nonsignificantly different values.

### Cytokine Analysis

Peripheral blood samples were collected in weeks 1, 2 and 7 by venous puncture and sera were stored at −80°C until analysis of cytokines. The analysis was performed on serum in which there was no hemolysis. The sera were thawed, spun (3000 rpm, 10 min) to remove debris and incubated with antibody-immobilized beads overnight at 2−8°C. Assays were run according to the manufacturer’s instructions using a human cytokine/chemokine/growth factor 47-plex panel and a Millipore kit for Luminex (Merck KGaA, Darmstadt, Germany). The following were analyzed: sCD40L, epidermal growth factor (EGF), eotaxin, fibroblast growth factor 2 (FGF-2), Fms-related tyrosine kinase 3 ligand (FLT-3L), fractalkine, granulocyte colony-stimulating factor (G-CSF), granulocyte-macrophage colony-stimulating factor (GM-CSF), growth-regulated oncogene) - alpha (GRO-α), IFN-α2, IFN-γ, IL-1α, IL-1β, IL-1RA, IL-2, IL-3, IL-4, IL-5, IL-6, IL-7, IL-8, IL-9, IL-10, IL-12 (p40), IL-12 (p70), IL-13, IL-15, IL-17A, IL-17E/IL-25, IL-17F, IL-18, IL-22, IL-27, IP-10, monocyte chemoattractant-1 (MCP-1), MCP-3, macrophage colony-stimulating factor (M-CSF), MDC, MIG, macrophage inflammatory protein 1α (MIP-1α), MIP-1β, platelet-derived growth factor (PDGF-AA), PDGF-AB/BB, TGF-α, tumor necrosis factor (TNF-α), TNF-β, vascular endothelial growth factor (VEGF-A). Measurements and data analyses were performed using the standard set of programs Magpix (Milliplex MAP). Serum LDH activity was analyzed using a kinetics method according to the manufacturer’s instructions (DDS *in vitro* Solutions, Pushchino, Russia).

Biochemical studies (glucose, creatinine, C-reactive protein, AST, ALT) were performed on an Indiko automatic biochemical analyzer (ТhermoScientific, Finland) using the manufacturer’s reagents. Control materials were produced by RANDOX (Randox Laboratories, UK). Coagulation parameters (fibrinogen, D-dimer) were analyzed on coagulation analyzer ACL TOP 500 (Instrumentation Laboratory, USA) using the manufacturer’s reagents.

### HMGB1 Analysis

Serum HMGB1 was assayed by using an ELISA kit according to the manufacturer’s instructions (IBL International, Hamburg, Germany).

### Dataset and Pre-Processing for Building AI Models

To build prediction models for COVID-19 severity, we considered 30 moderate and 30 severe cases. We chose only 19 cytokines and blood markers (MDC, glucose, creatinine, fibrinogen, CRP, IL-6, TNF-α, IL-8, MIP-1β, IL-18, MIG, IP-10, ALT, LDH, APTT, D-dimer, HMGB1, neutrophil counts, monocyte counts) for which the differences between controls and patients and/or between severe and moderate were statistically significant. We focused on the results of cytokines/blood tests carried out during the first days after hospitalization (week 1).

The resulting dataset was pre-processed for use of machine learning algorithms. The missing values were replaced with the average value for the respective group. Since most algorithms depend on data scaling, the data were normalized by z-score normalization, also known as standardization. The values of each attribute were transformed using the following formula $X'=\frac{x-\mu }{\sigma }$ , where *µ* and *σ* are the mean and the standard deviation of the feature values, respectively. To solve the problem of binary classification, patients with a moderate course of COVID-19 were assigned to class 0, and those who had severe disease to class 1.

### Prediction Algorithms

We used three classification algorithms to predict the severity of COVID-19. Logistic regression is a model in which the response is a categorical variable denoting a patient class (40). Logistic regression using a logistic function allows estimation of the probability of a binary response based on predictor variables.

Support Vector Machine, and in particular Support Vector Classifier, is an algorithm based on finding a hyperplane in a feature space that best separates data points belonging to different classes (41). The aim is to define the optimal hyperplane that has the maximum margin, *i.e*., the maximum distance between data points in the two classes. Margin maximization is performed so that future data points can be classified with more confidence.

Random forest is an ensemble of concurrently trained independent decision trees (42). Each individual tree from the ensemble predicts the class of a patient, and then the class with the most votes becomes the prediction of the Random Forest Classifier. Decision tree is a tree-structured classifier in which nodes represent certain decision rules, which allows splitting the feature space into parallelepipeds containing objects of only one class (43).

A cross-validation approach was used to select the model hyperparameters (the regularization parameter in logistic regression and support vector machine models, as well as the number of decision trees in random forest). Since the dataset is small and there is no way to divide it into two independent datasets for training and testing, then the cross-validation was used to develop predictive models and measure their performance. In particular, we used leave-one-out cross-validation approach, which is preferred for small datasets: machine learning algorithms are trained *N* times on *N -* 1 objects from the sample and then tested on the remaining one. Here, *N* is the total number of considered objects in the sample. The final performance measure is defined as the average of the values computed for each partition. The prediction abilities of the models were compared using the classification accuracy (ratio of true predictions to all predictions).

The above algorithms were implemented using Python v3.7.5 and *scikit-learn* package v0.23.1. The data were visualized by principal component analysis performed using R v4.0.2 with the *prcomp* function from *stats* package and *fviz_pca_biplot* function from *factoextra* package. Principal component analysis is a technique used to identify strong patterns in a dataset and transform high-dimensional data to low-dimensional data (2D or 3D) so that it can be visualized easily (44). The new subspace is defined to maximize data variability in the orthogonal projection onto the subspace.

#### Most Important Feature Selection

An advantage of the classification algorithms adopted here is that they quantify the importance of features. The larger this number, the greater the influence of the marker on the prediction of the target variable. If there is an evaluator that assigns weights to features, we can perform recursive features selection in order to leave the most significant markers. Additionally, the removal of some features from consideration can improve the classification accuracy. We used the recursive feature elimination strategy (*RFE* function from *scikit-learn* Python package) together with each classifier to eliminate the redundant features and to select the most important ones.

### Statistical Analysis

P values comparing moderate to severe cases are produced by comparison of the data between the two groups for normal (Gaussian) distribution (alpha = 0.05) using D’Agostino & Pearson test for the demographics and baseline characteristics of patients. The normally distributed data were analyzed by the unpaired parametric test, while unpaired non-parametric data were analyzed by Mann-Whiney test. The levels of inflammatory mediators, damage parameters and biochemical parameters in the sera of patients (severe, moderate and healthy controls) were analyzed by one-way ANOVA and Kruskal-Wallis test for multiple comparisons. The significance of the p-values are as follows: * p < 0.05; ** p < 0.01; *** p < 0.001; **** p < 0.0001. Following analysis for Gaussian distribution, Pearson correlation coefficient and two-tailed p values were calculated for the selected datasets using GraphPad Prism 9.0.

## Results

### Characteristics of the Patients

All the patients in the study were hospitalized with COVID-19. The diagnosis of COVID-19 and treatment were made according to the Ministry of Health of Russian Federation “Temporary guidelines on the prevention, diagnosis and treatment of COVID-19” version 7.0” (39). Their demographic characteristics are summarized in **Table 1**. The most common complaints were high fever, cough, muscle weakness, shortness of breath, anosmia, chest pain, headache, and muscle ache. Less common were throat pain and diarrhea. The patients were hospitalized for a median of 9.1 ± 6 days to 9 ± 3.6 days for severely and moderately ill patients, respectively. At admission, severely ill patients had a higher breathing rate than patients with moderate COVID-19. Peripheral capillary oxygen saturation (SpO_2_) reached 95.2 ± 1.9% in moderate cases and was lower in severe cases (89.9 ± 5.3%). Fifteen patients with severe COVID-19 received non-invasive oxygen support, while those with a moderate form of the disease did not require oxygen support.

Lung damage as assessed by computed tomography reached more than 75% in 27 out of 30 severely ill patients, and two more patients progressed to 75% lung damage in week two. One patient had 50% lung damage. Only 10% of the patients with moderate COVID-19 had 75% lung damage, 27% had about 25%, and in 63% lung damage exceeded 25%. Lesion volume in both lungs was scored on a semi-quantitative scale according the to the Russian national guidelines (45, 46) from CT-0 to CT-4 with a 25% step (CT-0: 0%, CT-1: 25%, CT-2: 50%, CT-3: 75%, CT-4: 100%). Half of the severely affected patients were admitted to the ICU for 9 ± 3.6 days. All four deceased patients were mechanically ventilated and died from acute respiratory distress syndrome and endotoxic shock.

Most of the severely affected COVID-19 patients had comorbidities (80%). The most common comorbidities in severely ill patients were arterial hypertension (80%), diabetes (43%), malignancy (16%), and chronic lung diseases (6.6%). However, only 60% of the moderate cases had comorbidities, and the most common was arterial hypertension (53%, **Table 1**). All patients had pneumonia with typical ground glass opacities in computed tomography (CT) scans of the lungs. Thromboembolic events occurred in 56% of the patients with severe disease but in only 16% of patients with moderate disease.

### Inflammatory Markers

**Figure 1:**
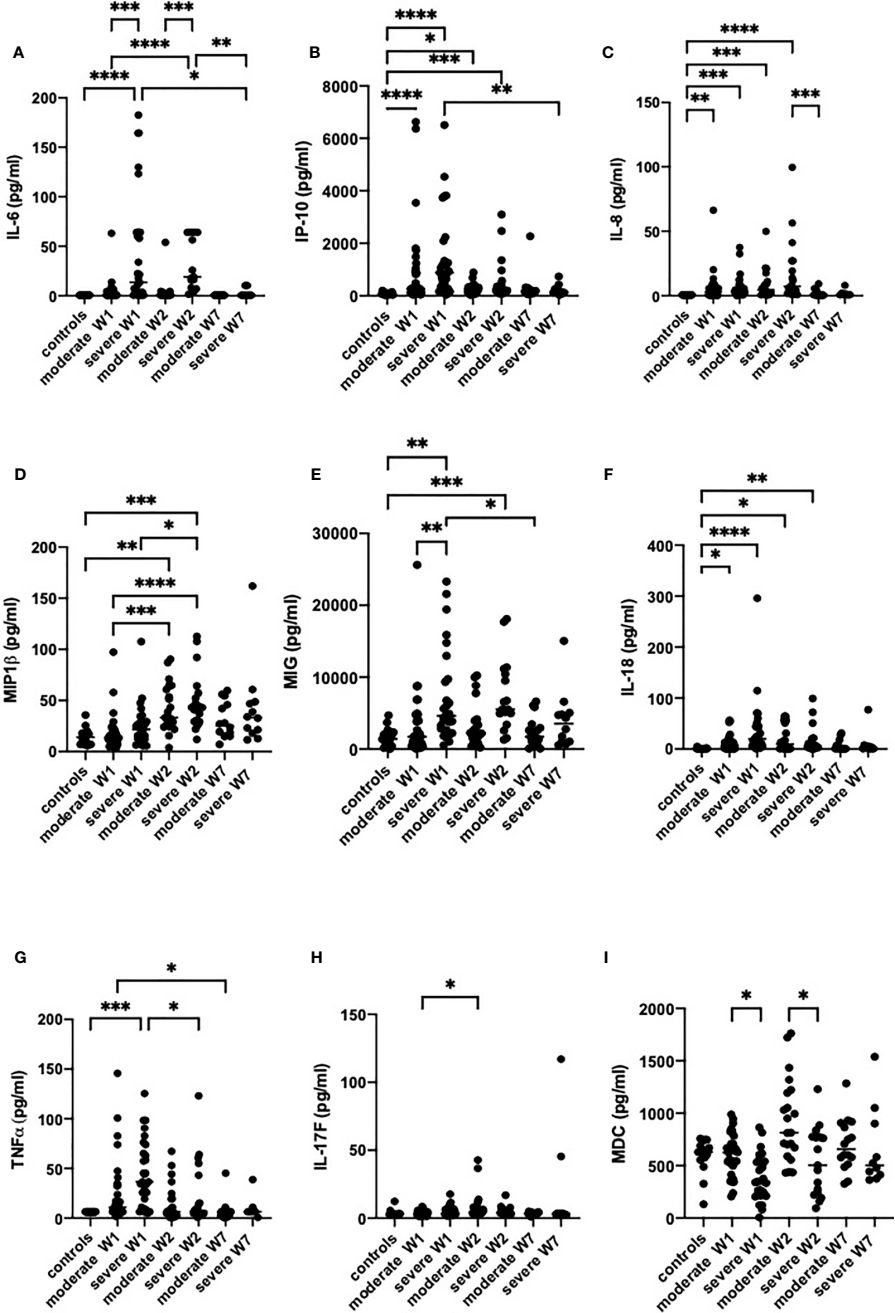
The levels of inflammatory mediators IL-6 **(A)**, IP-10 **(B)**, IL-8 **(C)**, MIP-1β **(D)**, MIG **(E)**, IL-18 **(F)**, TNF-α **(G)**, IL-17F **(H)** and MDC **(I)** in the sera of patients with moderate or severe COVID-19. Cytokines were analyzed in healthy controls (n = 17) and in moderate or severe COVID-19 cases in week 1 (W1), week 2 (W2) and week 7 (W7) by Luminex. The data from moderate COVID-19 cases in W1 (n = 30), W2 (n = 18) and W7 (n = 13), and in severe cases in W1 (n = 29), W2 (n = 15) and W7 (n = 8) are presented as scatter plots of each individual value with a line at the median. The data were analyzed by one-way ANOVA and Kruskal-Wallis test for multiple comparisons. *p < 0.05, **p < 0.01, ***p < 0.001, ****p < 0.0001.

The cytokine and chemokine levels were analyzed in serum of patients at admission, two weeks after admission, and in week seven. In agreement with previously published studies (17, 47–49), we observed a hyperinflammatory syndrome in severe cases, which required oxygen support. In serum of patients with severe COVID-19, the levels of IL-6, MIG, TNF-α, IL-8, IL-18 and IP-10 were higher than in the healthy controls (**Figures 1A** **–G**), while in moderate disease only the levels of IL-18, IL-8 and IP-10 were higher. At hospital admission, only three cytokines were different between the severe and moderate groups, namely IL-6, MIG and MDC. IL-6, a cytokine that was attributed mostly to the cytokine storm (48), was significantly increased in severe cases in weeks 1 and 2, while patients with a moderate form of the disease did not show an increase in IL-6 levels (**Figure 1A**). In week 1, MIG was lower in severely ill patients than in patients with moderate disease (**Figure 1E**). MDC was significantly lower in severe than in moderate disease in weeks 1 and 2. TNF-α levels were strongly increased in moderate and severe disease in week 1, but in week 7 it was at the detection limit. MIP-1β significantly increased in week 2 in both moderate and severe disease. IL-8 and IL-18 were increased in moderate and severe disease during weeks 1 and 2, but the levels were normalized in week 7. IP-10 was increased in both severe and moderate disease in week 1, it was higher in moderate disease in week 1, and normalized starting from week 2. In severe cases, the IP-10 levels remained high in week 2 but by week 7 it diminished to control values (**Figure 1B**).

IL-17 levels were not different between the two severity groups, with the exception of two patients with severe disease in whom IL-17 increased in week 2 and remained elevated in week 7 (**Figure 1H**).

Thus, the severely sick patients had higher levels of TNF-α, IL-6, IL-8, IL-18, MIG and IP-10 than the healthy controls, while in patients with moderate disease only IL-8, IP-10 and IL-18 were significantly higher than in healthy controls. In both groups, the cytokine levels returned to control levels by week 7. Noteworthy is that the levels of 18 of the 47 cytokines tested were above the detection limit. The levels of cytokines that were not different between the groups are presented in **Figure E1**.

### Organ Damage Markers

**Figure 2:**
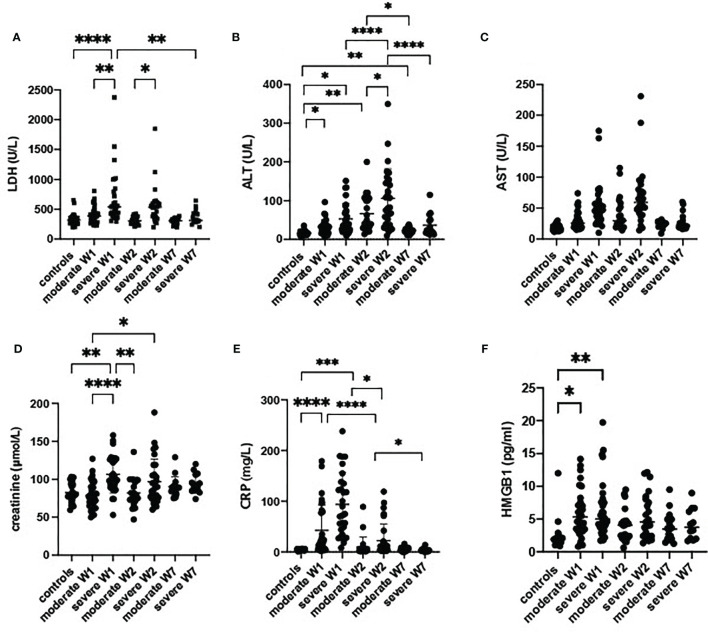
Damage parameters in serum of patients with a moderate or severe form of COVID-19. Lactate dehydrogenase (LDH) **(A)**, alanine aminotransferase (ALT) **(B)**, aspartate aminotransferase (AST) **(C)**, creatinin **(D)**, C reactive protein (CRP) **(E)** and high mobility group box 1 (HMGB1) **(F)** were analyzed in sera from controls (n = 17) moderate COVID-19 cases in W1 (n = 30), W2 (n = 18) and W7 (n = 13), severe cases in W1 (n = 29) and W2 (n = 15), and severe cases in W7 (n = 8) are presented as scatter plots of each individual value with a line at the median. The data were analyzed by one-way ANOVA and Kruskal-Wallis test for multiple comparisons. *p < 0.05, **p < 0.01, ***p < 0.001, ****p < 0.0001.

The classical organ damage parameters, including LDH and ALT, were significantly higher in weeks 1 and 2 in severe cases than in healthy controls, but were fully normalized in week 7 (**Figures 2A, B**). The levels of creatinine showed the same trend (**Figure 2D**). In contrast, HMGB1, a damage-associated molecular pattern, was also increased from week 1 but remained high in moderately and severely ill patients up to week 7 (**Figure 2F**). AST was not different between the groups (**Figure 2C**).

**Figure 3:**
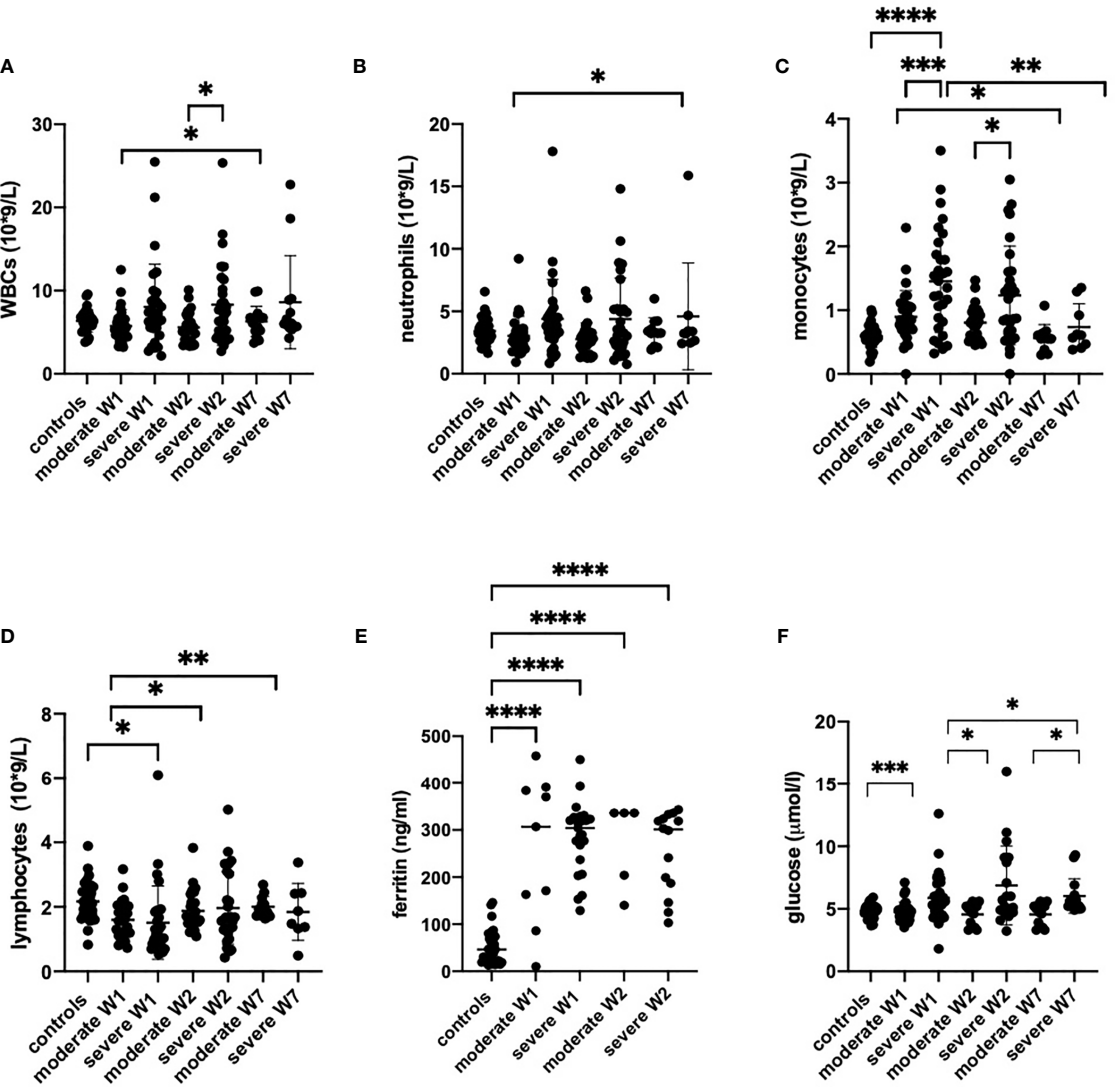
The peripheral blood counts **(A–D)**, serum ferritin **(E)** and glucose levels **(F)** from healthy controls (n = 17), moderate COVID-19 cases in week 1 (W1) (n = 30), week 2 (W2) (n = 18) and week (W7) (n = 13), and in severe cases in W1 (n = 29), W2 (n = 15) and W7 (n = 8) are presented as scatter plots of each individual value with a line at the median. The data were analyzed by one-way ANOVA and Kruskal-Wallis test for multiple comparisons. *p < 0.05, **p < 0.01, ***p < 0.001, ****p < 0.0001.

The CRP levels were high at admission and normalized by week 7 (**Figure 2E**). Notably, we observed a strong correlation of LDH with CRP (**Figures E2A, B**). In week 2, a correlation existed between LDH and HMGB1 (**Figure E2C**). Moreover, ferritin (**Figure 3E**), fibrinogen and D-dimer levels (**Table 2**) were higher in the severe cases than in moderate cases.

**Table 2:** The analysis of blood coagulation parameters in peripheral blood at admission (week 1) and at week 2.

	Controls	Week 1		Week 2	
		Moderate	Severe	Moderate	Severe
**APTT, sec**	28 ± 2,3	30,2 ± 3,9	32,7 ± 15	30,35 ± 3.92	32,8 ± 6,9
		ns	ns	***p = 0,0006	***p = 0,0006
**INR, units**	1,0 ± 0,1	1,12 ± 0,1	1,11 ± 0,1	1,12 ± 0,1	1,06 ± 0,1
		ns	ns	ns	ns
**0-dimer,ng/ml**	136 ± 50	752 ± 1165	1051 ± 20	306 ± 180	452 ± 229
		****p = < 0,0001	****p < 0,0001	**p = 0,0061	****p = < 0,0001
		**p = 0,0036	creatinine + *p = 0,0131		
**Anti-thrombin 3, %**	94 ± 17	98 ± 24	107 ± 20	98,38 ± 24,36	99 ± 14
		ns	**p = 0,0034	ns	ns
			*p = 0,0107		
**Prothrombin time, sec**	11,6 ± 0,43	12,52 ± 2	13,6 ± 1,9	12,5 ± 1,17	12,2 ± 1,37
		****p < 0,0001	****p < 0,0001	*p = 0,0145	
			**p = 0,0033		
			^§§§^p = 0,0006		
**Fibrinogen,g/L**	2,8 ± 0,5	4,56 ± 1,46	6,4 ± 1,8	4,52 ± 1,5	3,6 ± 1,2
		***p = 0,0005	****p = < 0,0001	ns	ns
		^####^p < 0,0001			
		^§^p = 0,0384	^§§§§^p < 0,0001		

The activated partial thromboplastin time (APTT), the international normalised ratio (INR), D-dimer, antithrombin-3, prothrombin, and fibrinogen were analyzed. Data are presented as mean ± standard deviation. The data were first analyzed for normal (Gaussian) distribution (alpha = 0.05) using D’Agostino & Pearson test. The normally distributed data were then analyzed by the one-way ANOVA with Dunnett’s multiple comparisons test. While the data that were not normally distributed were analyzed by an ANOVA Kruskal-Wallis test with Dunn’s test for multiple comparisons. The paired values between the same patients at week 1 and week 2 were analyzed by Wilcoxon matched -pairs signed rank test (&p < 0.05). The adjusted p values are provided, where the p values were less than < 0.05 compared to healthy controls (*), between the groups with different disease severity (#) and in the same patients tested at week 1 and week 2 (&), ns stands for nonsignificantly different values.

### Monocyte Activation in Severe COVID-19 Patients

The patients with moderate disease had normal counts of white blood (WBC), neutrophils and lymphocytes in their peripheral blood throughout the course of the disease (**Figure 3**). However, the monocytes were significantly upregulated from the first week in moderately ill patients but normalized by week 7. In severe cases, the numbers of WBC were increased during weeks 1 and 2 (**Figure 3A**) but returned to normal, with the exception of two patients who continued to have elevated WBC counts. Remarkably, monocytes were strongly increased in severely ill patients in weeks 1 and 2 but returned to normal in week 7 (**Figure 3C**). In severely ill patients, neutrophils increased steeply in weeks 1 and 2. One patient maintained high neutrophil count up to week 7 (**Figure 3B**). Again, the number of monocytes were strongly increased in weeks 1 and 2 in severe cases but returned to normal in week 7. The patients with severe disease have shown lymphopenia at week 1 while the lymphocyte numbers in moderate cases were comparable to controls.

**Figure 4:**
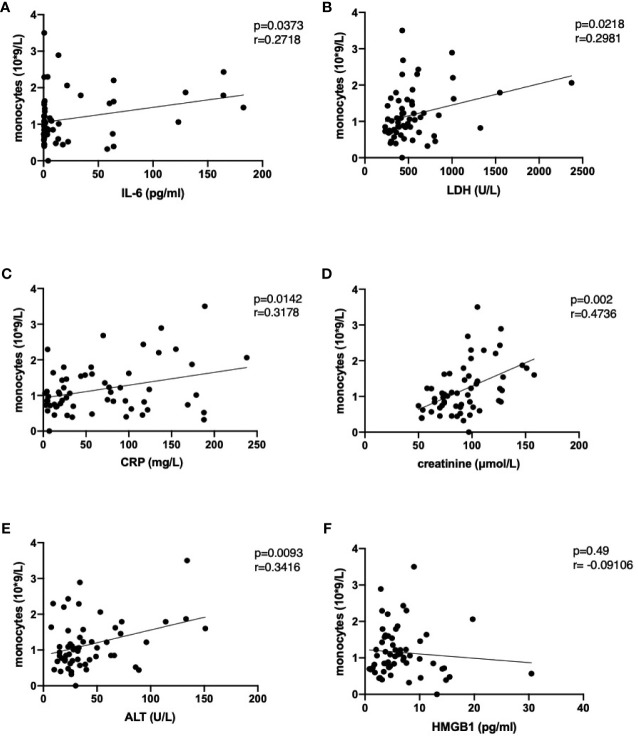
Correlation of monocyte numbers with selected damage parameters IL-6 **(A)**, lactate dehydrogenase **(B)**, C-reactive protein **(C)**, creatinine **(D)**, alanine aminotransferase **(E)** and high mobility group box 1 **(F)** in peripheral blood of COVID-19 patients. Following analysis for Gaussian distribution, Pearson correlation coefficient and two-tailed p values were calculated for the selected datasets using GraphPad Prism 9.0.

The increase in peripheral monocyte count in severe cases was accompanied by higher levels of IL-6, IL-8, MIG and IP-10. This could be indicative of the previously reported aberrant monocyte activation in severely ill patients (50). The number of monocytes in peripheral blood correlated with the IL-6 levels and with damage parameters such as CRP, LDH, ALT and creatinine (**Figure 4**) but no correlation was found between those parameters and neutrophil numbers (**Figure E3**). Of interest, although HMGB1 significantly increased in severely ill patients, it was not correlated with monocyte or neutrophil numbers (**Figures 4F**, **E3F**). The increase in peripheral monocyte numbers in the severe cases compared to the moderate cases was accompanied by higher IL-6 and MIG and significantly lower MDC levels in serum (**Figure 1**). The levels of MDC were significantly reduced in patients with severe COVID-19 compared to moderate disease during weeks 1 and 2. In week 2, patients with moderate disease showed induction of MDC in blood, while in severely affected patients it remained downregulated. All these features could be indicative of aberrant monocyte activation in severe COVID-19 disease.

### Artificial Intelligence Predicts Severity of COVID-19

We considered various machine learning algorithms to build a predictive model of the severity of COVID-19: logistic regression (LR), Support Vector Machine (SVM) and Random Forest (RF). First, we used grid search to find the best hyperparameters for classifiers. For the LR model, we determined the optimal regularization parameter (*C* = 0.014). The SVM model was configured with linear kernel and optimal regularization parameter (*C* = 0.035). The optimal number of decision trees in the RF ensemble was *n_estimators_* =48. We evaluated the constructed models using leave-one-out (LOO) cross-validation accuracy and obtained 82%, 83% and 82% for LR, SVM and RF, respectively. Thus, the severity of COVID-19 can be predicted with good accuracy on the basis of 19 markers.

**Figure 5:**
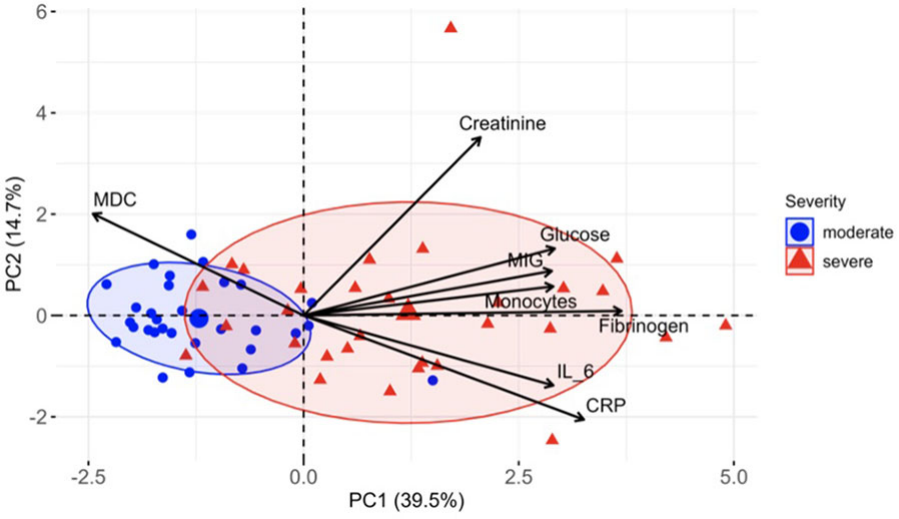
Distinct markers associated with COVID-19 severity. PCA of eight most important markers measured in COVID-19 patients at week 1. PC1 and PC2 explain 39.5% and 14.7% of the variation between patients, respectively, and segregate the patients by severity groups. Ellipses represent the 70% confidence interval of patient distribution in each group.

Further, to improve the performance of predictive models and determine the most important markers, we performed recursive feature elimination (RFE) with each classifier. As a result, based on the weights of the features assigned by the LR model, we identified a subset of 10 markers (**Table 3**, first column) for which the prediction accuracy calculated using the LR model was the highest of all subsets (83%). Using RFE together with SVM, we defined a subset of 10 features (**Table 3**, second column) for which the accuracy reaches a maximum of 87%. Finally, by performing feature selection based on the importance that the RF classifier calculated, we found an optimal subset of 12 markers (**Table 3**, third column) with a prediction accuracy of 85%. Eight important markers were highlighted by all three algorithms: MDC, fibrinogen, creatinine, glucose, MIG, monocytes, CRP and IL-6. The prediction accuracy using only these features was 83%, 85% and 80% for LR, SVM and RF, respectively. To visualize the space of the eight obtained features, we performed principal component analysis (PCA) among COVID-19 moderate and severe patients only, and projected the data on the first two principal components, PC1 and PC2. The data depicted in the plane of the principal components are shown in **Figure 5**. The results show that the group of severe cases was characterized by an increase in creatinine, glucose, MIG, monocytes, fibrinogen, IL-6 and CRP, and a decrease in MDC.

**Table 3:** The most important markers of COVID-19 severity identified by recursive feature elimination (RFE) in conjunction with machine learning algorithms such as Logistic Regression (LR), Support Vector Machine (SVM) and Random Forest (RF).

RFE + LRaccuracy = 83%	RFE + SVMaccuracy = 87%	RFE + RFaccuracy = 85%
MDC	MDC	Creatinine
Fibrinogen	Glucose	CRP
Creatinine	Creatinine	MIG
Glucose	IL-6	Monocytes
MIG	Fibrinogen	Fibrinogen
Monocytes	MIG	MDC
CRP	CRP	TNFa
IL-6	LDH	IL-6
LDH	AHTV	IL-18
TNFa	Monocytes	Glucose
		ALT
		D-dimer

In each column the markers are arranged in descending order of their importance, determined by the corresponding algorithm.

### Prediction Model Based on Logistic Regression

It is important to develop a convenient clinical decision model to predict the severity of COVID-19. To do this, we used a LR model that can provide a clear practical interpretation. According to this classification algorithm, we can calculate the value of the logistic function and determine the patient’s class:


$$f\left(x\right)=\frac{1}{1+e^{-x}}$$

where


$$\begin{array}{l} x=\beta_{0}+\beta_{1}\cdot MDC+\beta_{2}\cdot Fibrinogen+\beta_{3}\cdot Creatinine+\beta_{4}\cdot Glucose\\ \;+\beta_{5}\cdot MIG+\beta_{6}\cdot Monocytes+\beta_{7}\cdot CRP+\beta_{8}\cdot IL-6 \end{array}$$

is a linear combination of the values of 8 features under consideration.

The coefficients *β* were obtained from our trained LR model:


β0=0.0106657,β1=−0.15047358,β 2=0.12728877,β3=0.12279092,β4=0.10936836,β5=0.10746877,β6=0.1040643,β7=0.10321838,β8=0.09186946

Prediction of disease severity of a particular patient, with an average accuracy of 83% (the accuracy of the LR model), is based on the value of the logistic function. If *f*(*x*)>0.5, the patient has severe COVID-19; if *f*(*x*)<0.5, it is a moderate condition.

Thus, we have constructed a practical model for predicting the severity of COVID-19 based on eight cytokines/blood markers. This model could support clinicians’ decision-making and triage of COVID-19 patients. All the models we constructed enable accurate prediction of COVID-19 severity based on the values of eight factors as input, and as output classifying the patient as moderately ill (0) or severely sick (1).

## Discussion

Identifying patients who will develop a severe form of COVID-19 and multiple organ damage remains a puzzle. In this study, we analyzed tissue damage markers such as HMGB1, LDH, AST, ALT and blood coagulation parameters, as well as the cytokine profile, to identify a combination of parameters that would accurately foresee the development of severe forms of the disease. In our cohort of moderately and severely sick hospitalized COVID-19 patients, IL-6, MIG, TNF-α, IL-8, IL-18 and IP-10 were highest in patients with severe COVID-19.

Indeed, several studies have reported a significant increase in the levels of cytokines and chemokines in severe COVID-19 patients, including VEGF, hepatocyte growth factor, TNF-α, MIP 1-α, MCP-1, IP-10, IFN-γ, GM-CSF, G-CSF, M-CSF, IL-17, IL-13, IL-12, IL-10, IL-7, IL-6, IL-1, IL-2 and IL-4 (51). Interestingly, it has been reported that SARS-CoV-2 infection also elevates the secretion of anti-inflammatory cytokines (IL-4 and IL-10) (52). In our study, the levels of seven cytokines in COVID-19 patients (IL-6, IP-10, IL-8, MIG, MIP-1β, IL-18, TNF-α) were different from those in healthy controls, while M-CSF, MCP-1, FLT-3, VEGF, IL-1RA, EGF, eotaxin and MCP-1 were not different. Other cytokines (**Table E2**) remained below the detection limit in two independent measurements. In contrast to a study by Zhu et al., we did not find increased levels of anti-inflammatory cytokines such as IL-4 and IL-10 (9).

Increased IL-6 levels are an important hallmark of the cytokine storm, which is produced in response to infection and tissue damage. Several studies have correlated increased IL-6 levels with COVID-19 severity and mortality (53–55). Therefore, IL-6 was considered as an attractive target for the treatment of COVID-19 (56, 57). Moreover, there is a link between the hyperinflammatory syndrome and aberrant monocyte activation in COVID-19 patients, which was demonstrated by the dysregulated balance in monocyte populations with a preference for inflammatory CD14^+^ monocytes expressing *IL-1β*, *JUN, FOS, JUNB, KLF6, CCL4* and *CXCR4* in the circulation (58). Therefore, the circulating activated monocytes could further support the hyperinflammatory syndrome in COVID-19 patients (58). Importantly, in COVID-19 patients requiring ICU admission, significantly higher numbers of IL-6 producing monocytes have been reported (59). Surprisingly, in our study the levels of MDC were significantly lower in severely ill patients compared to patients with a moderate form of the disease. MDC signals through the CCR4 receptor and functions as a potent chemoattractant for Th2 lymphocytes, monocytes, monocyte-derived dendritic cells, and natural killer cells (60). It has been shown that MDC acts as a pro-inflammatory cytokine in cigarette smoke-induced pulmonary inflammation and sepsis (60). In the type-2 inflammatory response, especially in asthmatic patients, antigen exposure leads to up-regulation of the CCR4 ligands of MDC and TARC/CCL17 (61). Therefore, looking at all these findings together, in COVID-19 patients MDC could might act as a protective cytokine to counterbalance the massive type 1 biased inflammatory response.

We have shown that the damage parameters (*i.e.*, DAMPs) such as LDH have a strong correlation with CRP in COVID-19 patients. It should be noted that targeting HMGB1 and its receptor RAGE was considered an attractive treatment strategy for COVID-19 infection (62). Also, a recent study reported increased HMGB1 levels in serum of COVID-19 patients (63). The authors reported that at ICU admission, the plasma levels of HMGB1 and IL-6 correlated with D-dimer and C-reactive protein levels (28). However, in our study, despite a clear increase in HMGB1 levels in severe COVID-19, no correlation between HMGB1 and other parameters could be found. Moreover, our machine learning models did not show any prognostic value of HMGB1 in predicting the severity of COVID-19.

Multiorgan damage occurs in severe cases of COVID-19. For example, it has been recognized that early kidney injury is an important complication of COVID-19 and is accompanied by increased serum creatinine, hematuria and proteinuria. The kidney injury in severely ill patients was strongly associated with increased mortality (64). Moreover, several large studies in the USA have identified acute kidney injury in up to 50% of hospitalized COVID-19 patients (65). Thus, it is important to stress that multiorgan injury is the result of induction of a massive, regulated cell death associated with the release of several DAMPs, including HMGB1. Notably, HMGB1 is released from cells undergoing apoptosis (66), necroptosis (67) or ferroptosis (68). Thus, it is conceivable that in patients with severe COVID-19, multiorgan damage results from the induction of one or a combination of regulated cell death modalities. In this context, a case study has reported the presence of lipid peroxides, the major executors of ferroptosis (69), in the kidneys of a patient who died from COVID-19 (70). Massive apoptosis and necroptosis have also been shown in postmortem lung sections of deceased COVID-19 patients, and the cell death was associated with inflammatory cell infiltration and pulmonary interstitial fibrosis (71). The release of HMGB1 was increased in serum of patients with severe COVID-19 (54, 63), which is in line with the results of our cohort of patients.

It has been shown that COVID-19 is associated with activation of NLRP3 inflammasome and was linked to the more severe form of COVID-19 (72). IL-18 was correlated with markers of the severity of COVID-19, such as IL-6 and LDH (72). However, in our study, upregulation of IL-18 was observed in moderately and severely ill patients during weeks 1 and 2 but it returned to normal in week 7.

We investigated a dataset of confirmed COVID-19 patients collected from Nizhniy Novgorod, Russia and used machine-learning algorithms to predict the severity of the disease. We built three prediction models having an accuracy of over 80%. In addition, we identified eight important cytokines and blood markers that differentiate to a great extent severe from moderate disease.

Nevertheless, this study has the following limitations 1) the predictive models were constructed based on a relatively small sample size (60 patients) therefore the interpretation of our findings might be limited; 2) we only used leave-one-out cross-validation rather than external validation. However, this method was shown to be a valuable tool for building the prediction models (73). But despite this, the selected factors that allow determination of the severity of COVID-19 are consistent with those previously known in the literature and as more data become available, the whole procedure can easily be repeated to finetune the prediction models.

In summary, our study shows that exaggerated monocyte activation correlates with excessive organ damage hyperinflammatory syndrome and predicts the severity of COVID-19 by artificial intelligence with a precision of over 80%. Future studies should focus particularly on the practical clinical value of damage parameters, including developing a scoring system with plasma biomarkers for early recognition of COVID-19 patients at risk of developing severe disease. The use of the described prediction models across different clinical settings and populations will gain more insights into progression of COVID-19 disease.

## Data Availability Statement

The raw data supporting the conclusions of this article will be made available by the authors, without undue reservation.

## Ethics Statement

The studies involving human participants were reviewed and approved by Local ethics committee of Nizhny Novgorod State University, Nizhny Novgorod, Russia. The patients/participants provided their written informed consent to participate in this study.

## Author Contributions

OK designed and performed experiments, analyzed data and wrote the original draft. EGa and AB enrolled patients in the study, collected clinical information, and collected peripheral blood. OV and MI performed experiments related to machine learning and wrote the manuscript. EK and EGo performed experiments, analyzed data and revised the manuscript. CB revised the manuscript. MV and DK designed and supervised the study and revised the manuscript. All authors contributed to the article and approved the submitted version.

## Funding

The research was supported by the Ministry of Science and Higher Education of the Russian Federation, agreement No. 075-15-2020-808. DK group is supported by FWO-Flanders projects: FWO G043219N and FWO G016221N and by UGent BOF 01/O3618. OK and CB are supported by FWO 3G065319N.

## Conflict of Interest

The authors declare that the research was conducted in the absence of any commercial or financial relationships that could be construed as a potential conflict of interest.

## Publisher’s Note

All claims expressed in this article are solely those of the authors and do not necessarily represent those of their affiliated organizations, or those of the publisher, the editors and the reviewers. Any product that may be evaluated in this article, or claim that may be made by its manufacturer, is not guaranteed or endorsed by the publisher.

## Supplementary Material

Supplementary Figure 1 The levels of inflammatory mediators in the serum samples were analyzed in healthy controls (n = 19), and in moderate or severe cases of COVID-19 in week 1 (W1), week 2 (W2) and week 7 (W7) by Luminex. The data from moderate COVID-19 in W1 (n = 30), W2 (n = 18) and W7 (n = 13) and from severe COVID-19 in W1 (n = 29), W2 (n = 15) and W7 (n = 8) are presented as scatter dot plots of each individual value with a line at the median. The data were analyzed by one-way ANOVA and Kruskal-Wallis test for multiple comparisons.

Supplementary Figure 2 Correlation analysis of damage parameters in serum. The XY pairs were correlated between each other and the Pearson coefficient and a two-tailed p value were calculated for the selected datasets using GraphPad Prism 9.0.

Supplementary Figure 3 Correlation analysis of peripheral blood neutrophil counts and selected damage parameters. The XY pairs were correlated between each other and the Pearson coefficient and two-tailed p value were calculated for the selected datasets using GraphPad Prism 9.0.

Supplementary Table 1 Treatment options used for COVID-19 patients.

Supplementary Table 2 Summary of the detection limits for each parameter tested by Millipore kit for Luminex (Merck KGaA, Darmstadt, Germany).
