# Supplementary figures and images for: Artificial Intelligence Predicts Severity of COVID-19 Based on Correlation of Exaggerated Monocyte Activation, Excessive Organ Damage and Hyperinflammatory Syndrome: A Prospective Clinical Study
Source: Front Immunol. 2021 Aug 27;12:715072. doi: 10.3389/fimmu.2021.715072 (PMC8442605; doi:10.3389/fimmu.2021.715072)

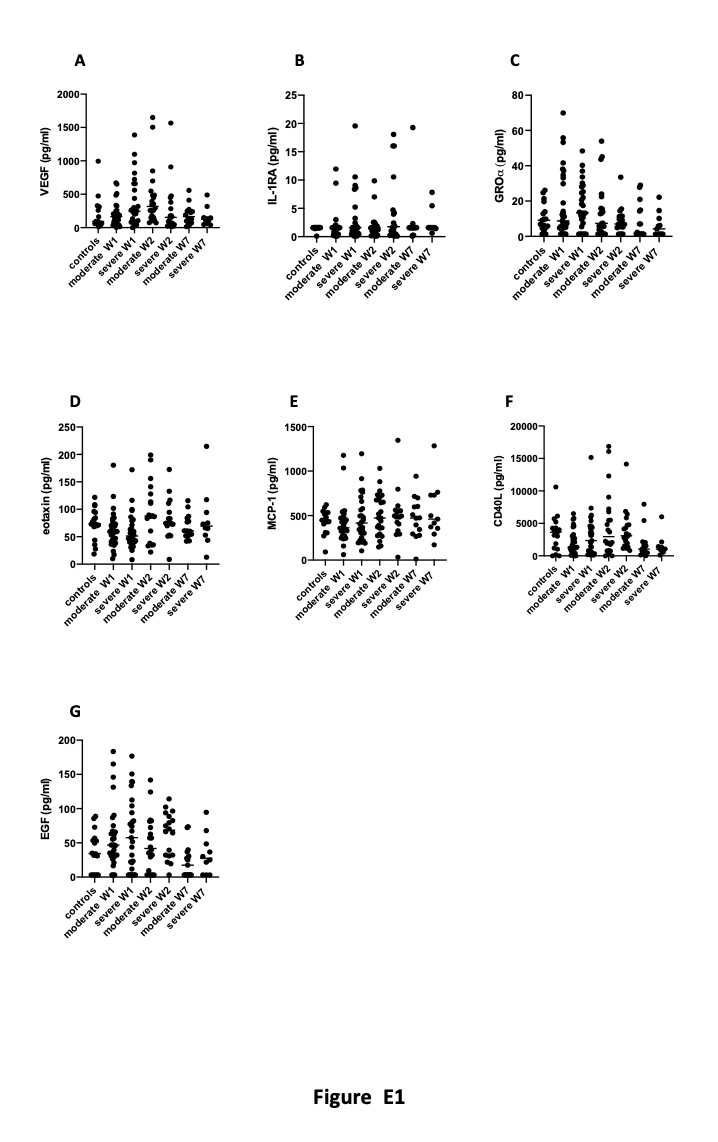

Supplement: Supplementary Figure 1 — The levels of inflammatory mediators in the serum samples were analyzed in healthy controls (n = 19), and in moderate or severe cases of COVID-19 in week 1 (W1), week 2 (W2) and week 7 (W7) by Luminex. The data from moderate COVID-19 in W1 (n = 30), W2 (n = 18) and W7 (n = 13) and from severe COVID-19 in W1 (n = 29), W2 (n = 15) and W7 (n = 8) are presented as scatter dot plots of each individual value with a line at the median. The data were analyzed by one-way ANOVA and Kruskal-Wallis test for multiple comparisons. [file Image_1.jpeg]

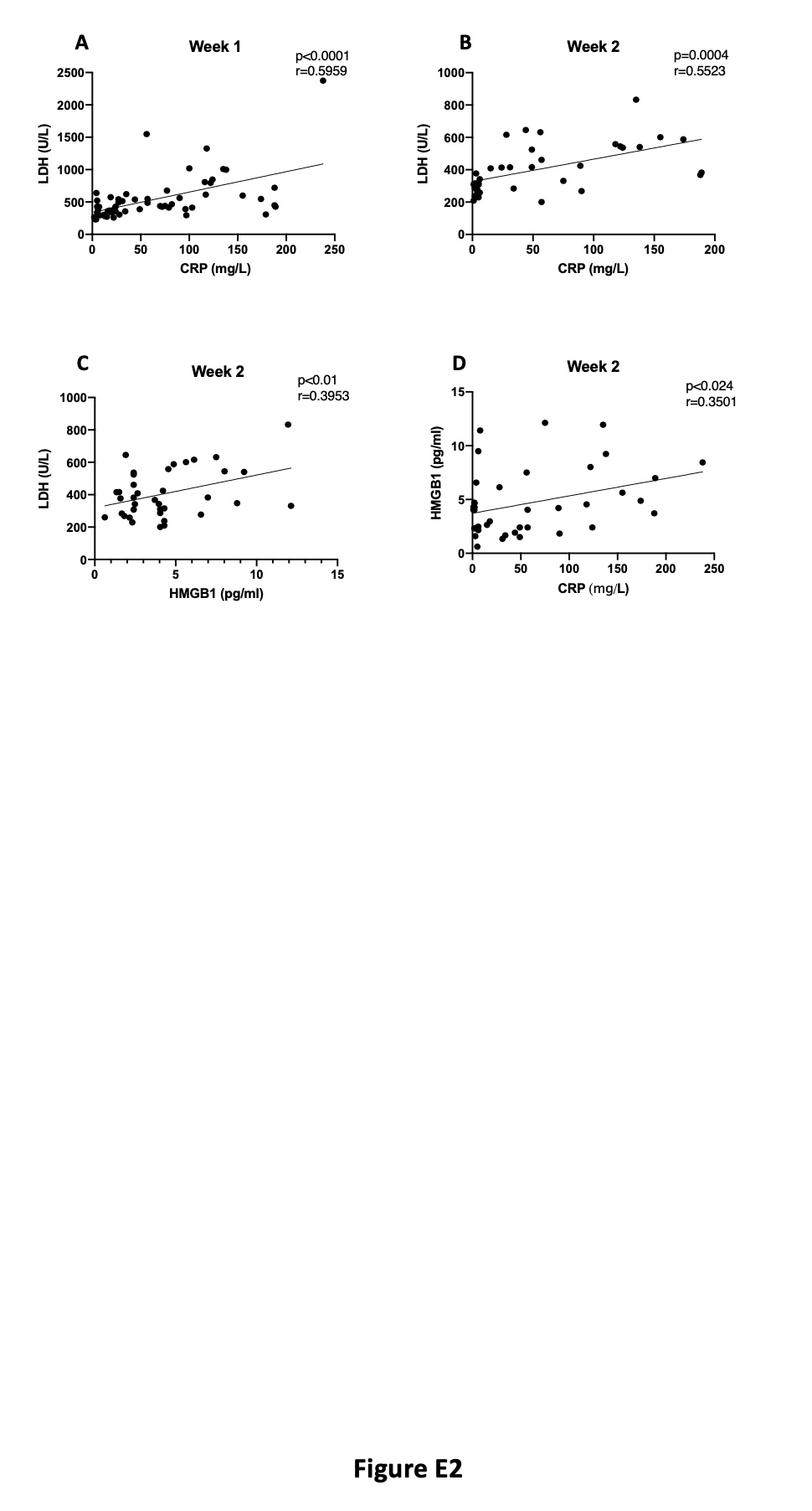

Supplement: Supplementary Figure 2 — Correlation analysis of damage parameters in serum. The XY pairs were correlated between each other and the Pearson coefficient and a two-tailed p value were calculated for the selected datasets using GraphPad Prism 9.0. [file Image_2.jpeg]

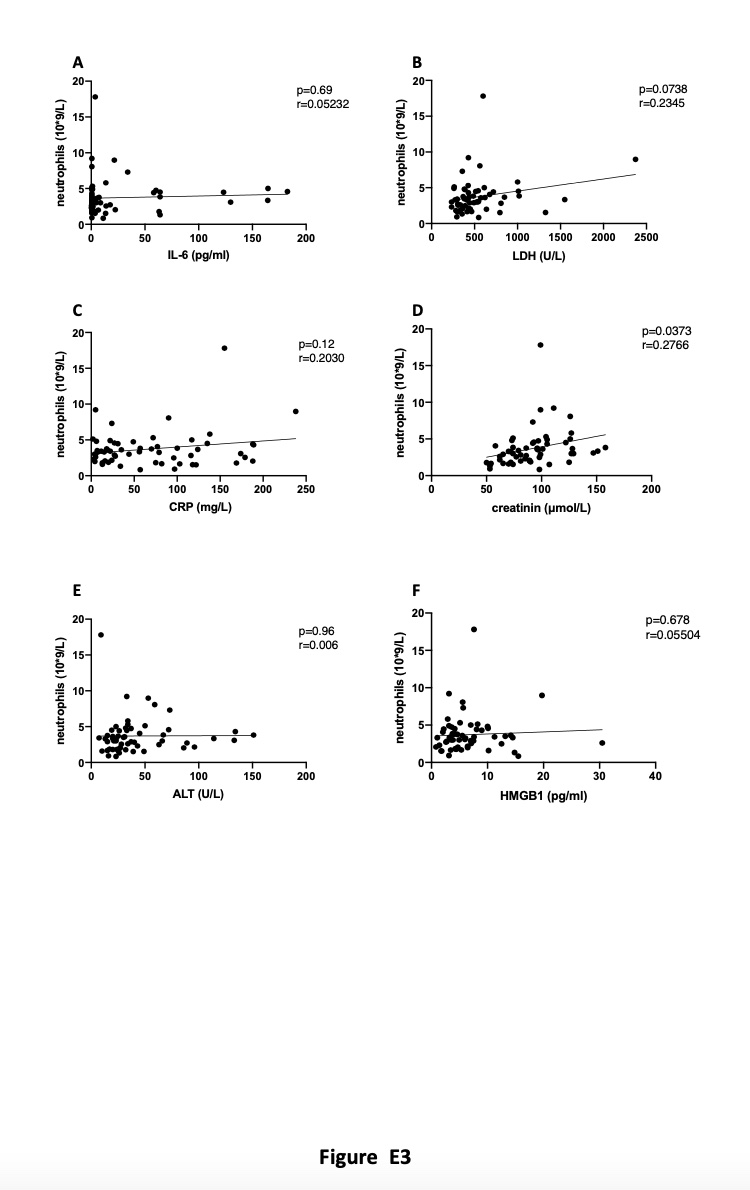

Supplement: Supplementary Figure 3 — Correlation analysis of peripheral blood neutrophil counts and selected damage parameters. The XY pairs were correlated between each other and the Pearson coefficient and two-tailed p value were calculated for the selected datasets using GraphPad Prism 9.0. [file Image_3.jpeg]
