# Supplementary material for: Artificial Intelligence Predicts Severity of COVID-19 Based on Correlation of Exaggerated Monocyte Activation, Excessive Organ Damage and Hyperinflammatory Syndrome: A Prospective Clinical Study
Source: Front Immunol. 2021 Aug 27;12:715072. doi: 10.3389/fimmu.2021.715072 (PMC8442605; doi:10.3389/fimmu.2021.715072)
Supplement: Supplementary Table 1 — Treatment options used for COVID-19 patients. [file Table_1.pdf]

Treatment	moderate	severe
Hydroxychloroquin	0	16
Azithromycin	0	15
Ceftriaxon	13	19
Amoxiclav	8	12
Dexamethasone, prednisolon	4	22
Levofloxacin	0	18
Ambroxol	19	22
Enoxaparin sodium	26	27
Umifenovir	8	8
Grippferon (IFNα-2β)	12	11
Tocilizumab (Acterma)	0	11
Calidavir (Calida)	0	8
Sarilumab (Kevzara)	1	1
Linezolid	0	2
Meropenem	0	1
Olokizumab	1	6
Avifavir	2	1
Basic therapy		
ACE inhibitors	15	17
β-blokkers	10	18
Statins	3	8
Insulin	1	9
Calcium channel blokkers	9	13

Table E1
