# Supplementary material for: Artificial Intelligence Predicts Severity of COVID-19 Based on Correlation of Exaggerated Monocyte Activation, Excessive Organ Damage and Hyperinflammatory Syndrome: A Prospective Clinical Study
Source: Front Immunol. 2021 Aug 27;12:715072. doi: 10.3389/fimmu.2021.715072 (PMC8442605; doi:10.3389/fimmu.2021.715072)
Supplement: Supplementary Table 2 — Summary of the detection limits for each parameter tested by Millipore kit for Luminex (Merck KGaA, Darmstadt, Germany). [file Table_2.pdf]

Cytokine	Detection limit (pg/ml)
CD40L	<12,8
EGF	<3,2
Eotaxin	<3,2
FGF-2	< 25,6
FLT-3L	< 0,96
Fraktalkine	< 32
G-CSF	< 4,8
GM-CSF	< 2,56
GROa	< 1,28
IFNa2	< 8
IFNy	< 1,28
IL-1a	< 4,8
IL-1b	< 1,6
IL-1RA	< 1,6
IL-2	< 0,64
IL-3	<1,28
IL-4	< 0,64
IL-5	< 0,64
IL-6	< 0,64
IL-7	<0,64
IL-8	<0,64
IL-9	< 0,64
IL-10	< 2,56
IL-12 (p40)	< 6,4
IL-12 (p70)	< 3,2
IL-13	< 6,4
IL-15	< 3,2
IL-17A	< 1,28
IL-17E/IL-25	< 40
IL-17F	<32
IL-18	< 0,64
IL-22	< 12,8
IL-27	<16
IP-10	<2,56
MCP-1	<3,2
MCP-3	< 8
M-CSF	< 40
MDC	<0,64
MIG	<6,4
MIP-1a	< 3,2
MIP-1b	<0,384
PDGF-AA	<12,8
PDGF-AB/BB	<9,6
TGFa	< 1,28
TNFa	< 6,4
TNFb	<1,6
VEGF-A	<2,6

Table E2
